# Novel *SZT2::MAST2* Fusion Detected in Salivary Duct Carcinoma

**DOI:** 10.1155/crip/3728015

**Published:** 2025-11-28

**Authors:** Adam Bedeir, Guilherme Rabinowits, Tolulope Adeyelu, Matthew J. Oberley, Mark G. Evans

**Affiliations:** ^1^Basis Phoenix High School, Phoenix, Arizona, USA; ^2^Department of Hematology and Oncology, Miami Cancer Institute/Baptist Health South Florida, Miami, Florida, USA; ^3^Department of Clinical and Translational Research, Caris Life Sciences, Phoenix, Arizona, USA; ^4^Department of Pathology, Caris Life Sciences, Phoenix, Arizona, USA

**Keywords:** case report, mast kinase fusion, salivary duct carcinoma, whole exome sequencing, whole transcriptome sequencing

## Abstract

Salivary duct carcinoma (SDC) is an uncommon neoplasm that often develops early regional and distant metastasis. Standard treatment for SDC is wide surgical resection along with lymph node dissection followed by adjuvant radiation therapy. The role of adjuvant chemotherapy is not clear with an overall low survival rate. SDC resembles high-grade invasive ductal carcinoma (IDC) of the breast both histologically and by immunohistochemistry (IHC). Fusions involving microtubule-associated serine/threonine (MAST) kinases have been suggested to play role in a subset of breast cancer, although the exact mechanism remains unclear. In this report, we present a case of SDC harboring a novel exon 1 of *SZT2* joined to exon 4 of *MAST2* leading to a likely pathogenic *SZT2::MAST2* fusion. Given the reported fusions involving the MAST kinases in breast cancer, this finding demonstrates an additional similarity between these two tumor types.

## 1. Introduction

Salivary duct carcinoma (SDC) is a rare yet highly aggressive malignancy. The estimated incidence is 1–1.2 in 1,000,000 patients, with a higher prevalence in men [1]. Based on its histological and immunohistochemical (IHC) features, IDC resembles invasive ductal carcinoma (IDC) of the breast [2]. The parotid gland is the most commonly involved salivary gland, accounting for approximately 80% of tumors, followed by the submandibular gland (8%–12%) [1, 2]. It is often diagnosed at an advanced stage, as it metastasizes early to regional lymph nodes and distant sites [3]. SDC is typically treated with wide surgical resection along with lymph node dissection followed by adjuvant radiation therapy. The role of adjuvant chemotherapy and targeted therapies is unclear to date [4].

Microtubule-associated serine/threonine (MAST) kinases are broadly expressed across various tissues, including the brain, heart, lung, liver, intestine, and kidney [5]. Malfunctions of MAST kinases are implicated in a wide range of diseases, including breast cancer [6–9]. Robinson et al. reported fusions involving *MAST1* or *MAST2* in breast cancer [6]. They also demonstrated that overexpression of MAST1 or MAST2 fusions products had a proliferative effect both *in vitro* and *in vivo*, suggesting a possible role in carcinogenesis in a subset of breast carcinomas.

To our knowledge, this is the first report of a case of SDC harboring a *SZT2::MAST2* fusion. Awareness of this fusion's occurrence in SDC contributes to the growing body of knowledge that may aid in the diagnosis and management of this rare and aggressive disease.

## 2. Case Presentation

**Figure 1:**
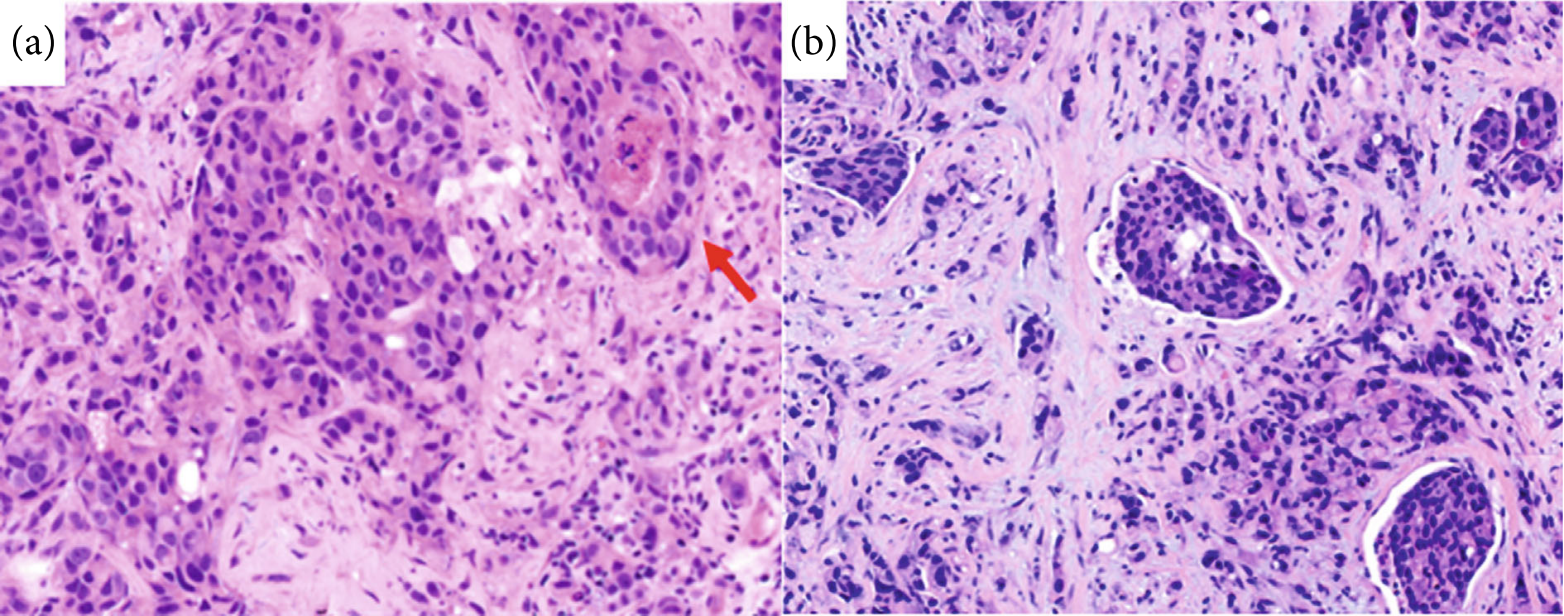
Histological features of the biopsied SDC ((a,b) hematoxylin and eosin staining, 40× magnification). (a) The tumor formed cords, nests, and cribriforming glands with central necrosis (arrow) infiltrating a desmoplastic stroma. The neoplastic cells showed ample eosinophilic cytoplasm and high-grade morphological features, including nuclear pleomorphism and frequent mitoses. (b) Micropapillae with stromal retraction and lacking fibrovascular cores were also appreciated.

**Figure 2:**
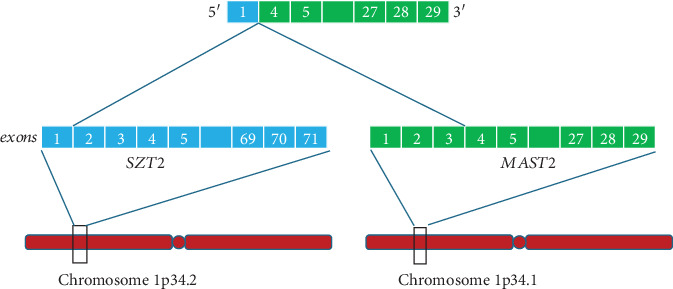
*SZT2::MAST2* fusion; exon 1 of *SZT2* is joined to exon 4 of *MAST2*.

**Figure 3:**
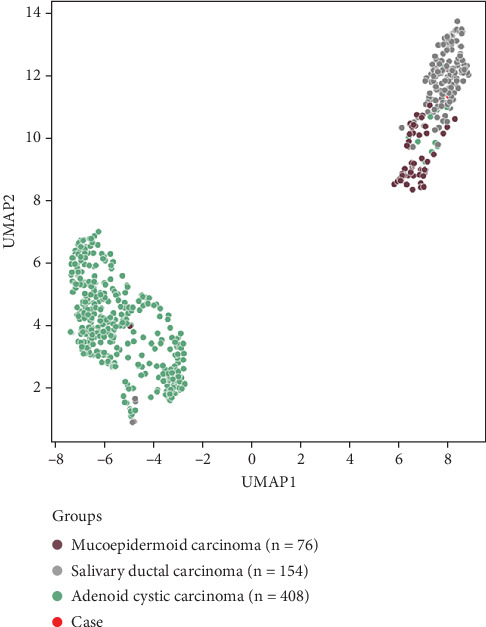
Uniform manifold approximation and projection (UMAP) of gene expression data in the biopsied tumor along with cases of salivary duct carcinoma, mucoepidermoid carcinoma, and adenoid cystic carcinoma.

A 59-year-old gentleman with no significant past medical history presented with an incidental 2-cm right parotid mass. Fine needle aspiration was not suggestive of a malignant disease. Seven years later, the patient developed throbbing pain in the right ear. On physical examination, a right parotid mass was identified and found to be nonmobile and tender. Neck MRI demonstrated a mass in the right parotid gland that had grown to 4.5 cm, as well as a prominent level 2 right carotid sheath lymph node measuring 14 mm, several enlarged posterior cervical chain nodes (largest 11 mm), and multiple abnormal nodes within the right supraclavicular fossa (largest 14.6, 13.7, and 11 mm). PET/CT imaging indicated a lytic bone lesion with intense FDG uptake (SUV max 11.8) in the right iliac crest, representing metastasis. Core biopsies of the parotid mass measuring 1.6 and 2.0 cm in length and 0.1 cm in diameter revealed an invasive tumor forming cords and nests within a desmoplastic stroma. The neoplastic cells showed ample eosinophilic cytoplasm and high-grade morphological features, including nuclear pleomorphism and frequent mitoses. Scattered cribriforming glands with central necrosis were identified (Figure 1A), as well as frequent nests without fibrovascular cores and demonstrating prominent stromal retraction (Figure 1B), consistent with the micropapillary subtype [10]. Immunohistochemistry (IHC) demonstrated that the tumor cells expressed CK7, GATA3, and androgen receptor, with focal GCDFP-15 expression, and no staining for HER2, estrogen receptor, mammaglobin, TTF-1, CDX2, p40, as well as melanocytic and neuroendocrine markers; an intraductal component was not demonstrated by p63 IHC. A high Ki67 proliferation index of 75% was observed. The diagnosis of SDC was made. A representative sample from the formalin-fixed parotid mass biopsy underwent whole exome sequencing, which detected the following pathogenic alterations: *TP53* p.S127P, *PIK3CA* p.E545K, *NF1* p. S1838fs, and *KMT2C* p.Q2539∗. Whole transcriptome sequencing (WTS) showed exon 1 of *SZT2* joined to exon 4 of *MAST2* leading to a likely pathogenic *SZT2::MAST2* fusion (Figure 2). Compared with gene expression data for previously tested cases of salivary gland cancers, specifically all subtypes of SDC, mucoepidermoid carcinoma, and adenoid cystic carcinoma, the patient's tumor was visualized with Uniform Manifold Approximation and Projection (UMAP) using K-means clustering (Figure 3), and a distinct association with SDC was observed.

The patient received 30 Gy palliative radiation over 15 fractions delivered to the parotid mass and neck lymph nodes, as well as 30 Gy over 10 fractions to the iliac lesion. He subsequently started bicalutamide, leuprolide, and alpelisib given the presence of a pathogenic *PIK3CA* alteration. Unfortunately, the patient was dismayed to learn that follow-up PET/CT imaging had indicated new osseous metastases of the L3 vertebral body (SUV max 4.7), T11 vertebral body (SUV max 4.0), and right clavicle (SUV max 2.8) that demonstrated the same lytic appearance as his original iliac crest lesion.

## 3. Discussion

SDC is a rare disease, accounting for approximately 1%–3% of all malignant salivary gland tumors [1]. However, it is a highly aggressive malignancy with early regional and distant metastasis. Local disease recurrence has been reported in 48% of patients at 17.4 months after initial treatment and distant metastases in 48% after an average of 28 months [11]. The most common sites of distant metastasis are the lung, bone, brain, and liver.

SDC shares many features with breast IDC. This is particularly important for the distinction between these two entities in metastasis to distant sites. SDC morphologically resembles a subset of IDC of the breast with higher Nottingham histological grade [1]. Both IDC and SDC are commonly composed of cells with ample eosinophilic cytoplasm that are arranged in nests, ducts, cribriform glands, and infiltrating cords in a desmoplastic stroma. In addition to morphological resemblance, they both show significant similarities in their IHC expression profiles. This includes positive staining for GATA3, GCDFP-15, and androgen receptor [1]. However, SDC is typically negative for estrogen receptor and progesterone receptor, which can help in this distinction; otherwise, morphological, clinical, and radiological correlations are necessary.

Currently, no National Comprehensive Cancer Network (NCCN) guidelines for the specific treatment of SDC exist. Similar to other salivary gland cancers, SDC is typically treated by surgery and postoperative radiotherapy [4]. Targeted therapy is often attempted in SDC treatment. Ninety percent of SDC cases show androgen receptor expression, making the tumor a potential target for androgen deprivation therapy [2]. HER2 expression occurs in between 15% and 40% of the cases, and 3+ IHC staining for HER2 has been associated with the early development of distant metastasis and poor outcomes [12]. Unfortunately, the overall survival rate for SDC remains low: the five-year survival rate for Stage I disease is 42%, for Stage II is 40%, for Stage III is 30.8%, and Stage IV is 23.2% [11]. This may in part be due to the rarity of this entity, as well as relative lack of understanding of the molecular alterations that play a role in its pathogenesis.

The molecular characteristics of SDC are heterogeneous. Common abnormalities include *ERBB2* amplification, as well various hotspot mutations involving *TP53*, *PIK3CA*, *HRAS*, and *PTEN* [13–20]. The current case did not show any significant copy number alterations; however, mutations in *TP53* and *PIK3CA* were detected. Multiple gene fusions have also been described in SDC including *BCL6::TRADD* and *ABL1::PPP2R2C* [13–18]. Additionally, fusions observed in other subtypes of salivary gland carcinoma, such as *ETV6::NTRK3* and *NCOA4::RET* described in secretory carcinoma and intraductal carcinoma, respectively [18, 19], have been reported in SDC without any histologic evidence of these other tumor types [13, 17]. To our knowledge, the presence of SDC subtype-specific genetic changes has not been documented. In the current case, other than *SZT2::MAST2*, no other fusion was identified. The overall findings are summarized in Table 1.

Protein kinases are critical for cellular signaling pathways, including those responsible for cell cycle, proliferation, and survival, signal transduction, metabolism, and gene expression [20]. The kinases mainly function by adding phosphoryl groups onto other substrates to influence their activity and/or stability. The catalytic activity of protein kinases resides within a kinase domain. [21]. MAST kinase family genes are characterized by the presence of a serine/threonine kinase domain, a 3⁣′ MAST domain with some similarity to kinase domains, and a PDZ domain [5, 21]. To our knowledge, the *SZT2::MAST2* fusion detected in the current report of SDC has not been previously described. However, this fusion is in-frame, and the protein is predicted to have an intact MAST2 kinase domain, suggesting a possible role in altering signaling pathways or cellular functions.

Although the exact mechanism is not clear, recurrent fusions of MAST kinases have been described in breast cancer. Robinson et al. described five functionally recurrent rearrangements of *MAST* in a cohort of about 100 breast cancer samples and 40 cell lines [6]. Three gene partners were found fused with the *MAST1* gene (*ZNF700*, *TADA2A*, and *NFIX*), and two were discovered in association with the *MAST2* gene (with *ARID1A* and *GPBP1L1*). All five *MAST* fusions encoded contiguous open reading frames, some retaining the serine/threonine kinase domain, with all retaining the PDZ domain and the 3⁣′ kinase-like domain. They also demonstrated that overexpression of these fused gene products led to enhanced proliferation in a benign breast cell line and that RNAi-mediated knockdown of MAST2 in a cancer cell line with *MAST2* gene fusions led to reduced growth and reduced tumor formation in mice xenografts. Their results suggested that MAST kinase family fusions occur in a subset of 3%–5% of breast cancers; however, to our knowledge, detailed clinical and pathological information about cancers harboring these fusions has not been previously documented. In this report, we present a case of SDC harboring a novel exon 1 of *SZT2* joined to exon 4 of *MAST2* leading to a likely pathogenic *SZT2::MAST2* fusion. Given the resemblance between both entities, it is possible that the current *SZT2::MAST2* fusion plays a similar role in the carcinogenesis of SDC, and its contribution to the pathogenesis of salivary tumors similar to our patient's warrants further investigation.

## 4. Conclusion

We report a case of SDC harboring a novel *SZT2::MAST2* fusion. It is similar to other reported fusions involving the MAST kinases identified in a subset of breast cancer. This finding highlights the potential role of whole transcriptome sequencing in aiding our understanding of the pathogenesis, diagnosis, and potentially management of the rare and aggressive disease that is SDC.

## Figures and Tables

**Table 1:** Common genetic alterations reported in SDC and those detected in the current case.

**Pathway**	**Genes Reported as Altered in SDC**	**Genes Altered in Current Case**
DNA damage	*TP53*, *ATM*	*TP53*
MAPK	*HRAS*, *BRAF*, *KRAS*, *NRAS*	*NF1*
PI3K	*PIK3CA*, *PTEN*	*PIK3CA*
Amplification	*ERBB2*, *NOTCH1*, *AKT1*, *SMARCA4*	None detected
Gene fusions	*ETV6::NTRK*, *BCL6::TRADD*, *ABL1::PPP2R2C*, *NCOA4::RET*	*SZT2::MAST2*

Abbreviations: MAPK, mitogen-activated protein kinases; PI3K, phosphoinositide 3-kinase.

## Data Availability

The data that support the findings of this study are available on request from the corresponding author. The data are not publicly available due to privacy or ethical restrictions.
